# Factors associated with postoperative nausea and vomiting after laparoscopic cholecystectomy at the National Referral Hospital, Bhutan: a cross-sectional study

**DOI:** 10.1186/s12871-024-02602-w

**Published:** 2024-07-22

**Authors:** Pema Jamtsho, Yeshey Dorjey, Namkha Dorji, Sangay Tshering, Kuenza P. Wangmo, Thinley Dorji, Tashi Wangchuk, Jampel Tshering

**Affiliations:** 1 Department of Anesthesiology, Jigme Dorji Wangchuck National Referral Hospital, Thimphu, Bhutan; 2 Department of Obstetrics and Gynaecology, Phuntsholing General Hospital, Chukha, Bhutan; 3 Department of Obstetrics and Gynaecology, Jigme Dorji Wangchuck National Referral Hospital, Thimphu, Bhutan; 4 Department of Internal Medicine, Central Regional Referral Hospital, Gelephu, Bhutan

**Keywords:** Anesthesia, Antiemetics, Cholelithiasis, Minimally invasive surgical procedures, Morbidity, Patient outcome assessment

## Abstract

**Introduction:**

Postoperative nausea and vomiting (PONV) are common distressing symptoms experienced after laparoscopic cholecystectomy. We report the rate, and the factors associated with postoperative nausea and vomiting, the patterns of prophylactic antiemetic prescription, and the anesthetic techniques used among patients who underwent laparoscopic cholecystectomy at the Jigme Dorji Wangchuck (JDW) National Referral Hospital, Bhutan.

**Methods:**

A cross-sectional study was conducted at the JDW National Referral Hospital, from January to December 2018. All the patients who underwent laparoscopic cholecystectomy under general anesthesia were included in the study. The demographic variables, premedication, induction agents, muscle relaxants, inhalational agents for maintenance, opioid and adjuvant analgesics, the reversal agents used, and the occurrence of PONV within 24 h were recorded. Data were analyzed using SPSS (version 23). *C*ontinuous variables were compared using a t-test or Mann-Whitney test, categorical variables were tested using chi-square or Fisher’s exact tests. Binary logistic regression analysis was performed to determine the factors associated with postoperative nausea and vomiting.

**Results:**

190 patients underwent laparoscopic cholecystectomy under general anesthesia. The rate of PONV after laparoscopic cholecystectomy was 31.1% (59/190). Over half (53.7%, 102/190) of the study population were within 21–40 years of age, over 80% (157/190) were female, and 2/3rd were overweight and obese. The most frequently used premedication was ranitidine (39%, 34/87) and metoclopramide (31%, 27/87). More than half (57.4%, 109/190) of the patients received morphine as an opioid analgesic before induction. Sodium thiopentone was a commonly used induction agent (65.8%, 125/190). Succinylcholine and atracurium were mostly preferred muscle relaxants. Isoflurane and air were the most used inhalational anesthetic agents for the maintenance of anesthesia. Ondansetron was the most preferred anti-emetics during the intraoperative period. Previous history of motion sickness (OR 5.8, 95%CI 2.9–11.2, *p* < 0.001), and use of sodium thiopental (OR 4.1, 95%CI 1.9–9.1, *p* < 0.001) were independent risk factors for PONV. The use of antiemetics (OR 0.1, 95%CI 0.0-0.4, *p* = 0.002), propofol (OR 0.2, 95%CI 0.1–0.5, *p* < 0.001), adjuvant analgesic paracetamol (OR 0.4, 95%CI 0.2–0.8, *p* = 0.026), and adequate hydration with IV fluids (OR 0.9, 95%CI 0.9-1.0, *p* = 0.042) were preventive factors for PONV.

**Conclusion:**

The rate of PONV after laparoscopic cholecystectomy was high. History of motion sickness and use of sodium thiopental for induction were independent risk factors of PONV. The use of multimodal prophylactic antiemetics was robust and superior to monotherapy in preventing PONV. This finding re-emphasizes the need for risk stratification and appropriate use of antiemetics and anesthetic agents to prevent PONV.

**Supplementary Information:**

The online version contains supplementary material available at 10.1186/s12871-024-02602-w.

## Introduction

Postoperative nausea and vomiting (PONV) are common complaints among patients undergoing surgery under general anesthesia [1]. A systemic review and meta-analysis with 22,683 patients who underwent laparoscopic cholecystectomy under general anesthesia from 11 countries showed a prevalence of PONV of 27.7% [2].

There are several risk factors for PONV including patient factors, anesthesia, and surgical factors [3, 4]. Patient factors are female gender, patient with a history of motion sickness, previous history of PONV, non-smokers, and prolonged fasting [5]. Anesthesia techniques are risk factors for PONV which includes, intra-operative use of opioids, nitrous oxide, volatile inhalational anesthetics, and some intravenous anesthetics (ketamine and etomidate). Types of surgery performed are also risk factors for PONV which include, gynecological surgery, ENT, breast surgery, laparotomy, craniotomy, and laparoscopic surgeries [3].

The causes of PONV after laparoscopic cholecystectomy are believed to be due to rapid peritoneal distension, activated neurogenic pathways by reaction reflexes, and splanchnic pressure and manipulations [6]. The creation of pneumo-peritoneum is an essential part of laparoscopy, leading to stretching of mechano-receptors, increased serotonin (5HT) synthesis, and PONV [7].

There are several strategies to prevent PONV which include non-pharmacological and pharmacological methods. Acustimulation including acupuncture and acupressure are non-pharmacological methods that are as efficacious as pharmacological agents in preventing PONV. The use of ginger, chewing gum, and carbohydrate loading are also found to prevent PONV [5]. Early and liberal resumption of oral intakes in the postoperative period is found to reduce PONV [5]. Various medications have been used to prevent PONV which includes, 5-HT3 antagonist (ondansetron), dopamine antagonist (metoclopramide), and corticosteroids (dexamethasone).

The prevention of PONV in high-risk patients significantly improves postoperative ratings of well-being and satisfaction [8]. Although the experience of PONV is generally self-limited, it can lead to rare but serious medical complications, such as aspiration of gastric contents, suture dehiscence, oesophageal rupture, subcutaneous emphysema, or pneumothorax [9, 10]. PONV may delay a patient’s discharge from Post Anaesthetic Care Units (PACUs) and can be the leading cause of unexpected hospital admission after ambulatory anesthesia [11].

In Bhutan, laparoscopic cholecystectomy is the most common surgical procedure performed. Over the years, the proportion of laparoscopic surgeries performed at JDW National Referral Hospital increased from 14% in 2019 to 22% in 2022 [12]. However, there is no study conducted on PONV after laparoscopic cholecystectomy in our setting. This study was conducted to determine the rate of PONV, the factors associated with PONV, and the patterns of prophylactic antiemetic prescription and the anesthetic techniques used in patients who underwent laparoscopic cholecystectomy at the JDW National Referral Hospital, Bhutan.

## Method

### Study design

This was an observational cross-sectional study conducted at the JDW National Referral Hospital, Bhutan from 1st January 2018 to 31 December 2018.

### Study setting

Bhutan is situated in the eastern Himalayas with a total population of over 0.77 million. The JDW National Referral Hospital is a teaching hospital located in Thimphu, the capital of Bhutan. It is a 380-bed hospital, and the Surgery Ward has 36 beds with 8 surgeons performing laparoscopic surgeries. The operation theatre has 8 rooms with 9 anesthesiologists and 11 nurse anesthetists.

Laparoscopic cholecystectomy is performed under general anesthesia and administered following a standard technique.

### Premedication

Prior to the induction of anesthesia, premedication is administered. Premedications commonly administered are midazolam (for anxious patients), ranitidine, and metoclopramide (for patients with a history of gastroesophageal reflux disease). Opioid analgesics morphine or fentanyl is administered before induction of anesthesia.

### Induction of anesthesia

For the induction of anesthesia, sodium thiopentone is commonly used. Propofol and ketamine are other induction agents that are in use besides sodium thiopentone.

### Muscle relaxants

Short-acting muscle relaxant succinylcholine is commonly used for endotracheal intubation. Other relaxants atracurium and vecuronium are used for the maintenance of muscles in a relaxed state for a longer duration.

### Maintenance of anesthesia

Inhalation anesthetic agents are used for the maintenance of anesthesia. The inhalation anesthetic agents used are either oxygen with nitrous oxide, or oxygen with air. Other inhalational agents often used are isoflurane, sevoflurane, and halothane.

### Adjuvant analgesics used per-operative

Inj. paracetamol (intravenous) is administered through infusion and often Inj. diclofenac sodium (intramuscular) is used as an adjuvant analgesic during the per-operative period.

### Antiemetics used per-operative

To prevent PONV, different types of antiemetic agents are used. The commonly used antiemetics are ondansetron, metoclopramide, and dexamethasone. Often these agents are used in combination (multimodal therapy) or singly (monotherapy).

### Reversal agents

Neostigmine and atropine are used for the reversal of anesthesia after completion of the surgical procedure.

### Postoperative analgesic medication

At the end of the surgery and before the reversal, surgical wound infiltration with local anesthetic agent with lidocaine is performed routinely as a part of postoperative analgesia. In the postoperative period, commonly used analgesics are Inj. tramadol, Inj. diclofenac sodium and Inj. paracetamol. These analgesics are used singly or in combination depending on the choices of the operating surgeon.

The types and combinations of agents used for premedication, induction, muscle relaxation, and maintenance of anesthesia are at the discretion of the anesthesia personnel attending the surgery. After achieving anesthesia, the start time of surgery is recorded corresponding to the skin incision given for the primary laparoscopic port, and the end of surgery is also noted corresponding to the end of skin closure time. During the intraoperative period, antiemetics and intravenous (IV) fluid are used in addition to the maintenance of anesthesia. The agents used for premedication, induction, muscle relaxation and maintenance, and IV fluid types and amount used intraoperative period are all recorded in an anesthetic monitoring chart. After completion of the surgery and extubation, patients are transferred to PACU and kept under close monitoring. After full recovery from anesthesia and clinically stable, patients are shifted back to the Surgery Ward. While in the ward, the patients are kept under close monitoring of vital signs and for any postoperative complications. The nurses on duty record the time when the patient compliant of nausea and or vomiting in PACU and in the ward during the first 24 h of the postoperative period. Rescue antiemetic with ondansetron or metoclopramide is administered for patients with PONV.

### Study population

All the patients who underwent laparoscopic cholecystectomy under general anesthesia at JDW National Referral Hospital during the study period were selected for the study.

### Inclusion criteria

Patients aged ≥ 18 years, with American Society of Anesthesiologists physical classification (ASA) status I, and ASA status II who underwent laparoscopic cholecystectomy under general anesthesia, and consented to participate were included in the study.

### Exclusion criteria

Patients of age < 18 years, patients with signs and symptoms of gastrointestinal, renal, and liver diseases; addicted to opioid drugs and alcohol, pregnant women, or breastfeeding; taking long-term corticosteroids; laparoscopy converted to open cholecystectomy; ASA status > II; and with cardiac arrhythmia or conduction abnormalities were excluded from the study.

### Primary outcome

The primary outcome was to determine the rate and the factors associated with PONV in patients who underwent laparoscopic cholecystectomy under general anesthesia.

### Secondary outcome

The secondary outcomes include the pattern of prescription of prophylactic anti-emetics, the anesthetic techniques used (premedication, anesthesia for induction, use of muscle relaxants, maintenance of anesthesia, and the reversal), intraoperative use of IV fluids, intraoperative and postoperative use of analgesics (opioid and adjuvant), and correlation of duration of surgery with time to compliant of PONV.

### Sample determination and sampling method

The sample size was determined using Cochran’s formula for a single population proportion considering the following assumptions: 95% confidence interval (Z = 1.96), and 5% margin of error (e = 0.05). The prevalence (p) of PONV is unknown in Bhutan, however, data from systemic review and meta-analysis showed the prevalence of PONV of 6.7–31.4% [2]. Therefore, at least 15%% prevalence of PONV is expected in Bhutan (*p* = 0.15); and q = 1-p.

$$\text{n}=\frac{{z}^{2}pq}{{e}^{2}}$$

$$\text{n}=\frac{{1.96}^{2}X 0.15 (1-0.15)}{{0.05}^{2}}$$

The final sample size (n) determined was 195. Convenience sampling was performed.

### Data collection tools and procedures

The pro forma developed for the study was piloted on 10 patients who were not included in the study. Written informed consent was obtained from all the study participants.

Demographic characteristics including age, gender, BMI, ASA physical status classification, previous history of surgery, history of PONV, history of motion sickness, history of gastritis and peptic ulcer disease, use of premedication, anesthetic, anti-emetics, analgesic agents, intraoperative use of IV fluids, duration of surgery, time to compliant PONV and postoperative events were recorded in a standard pro forma developed for the study (*supplementary file*).

### Operational definitions

#### Body mass index (BMI):

BMI is an index calculated using a height and weight of a person to provide an estimate of body fat in males and females of any age. BMI calculation is shown.

$$\text{B}\text{M}\text{I}=\frac{\text{w}\text{e}\text{i}\text{g}\text{h}\text{t} \left(\text{k}\text{g}\right)}{{Height \left(m\right)}^{2}}$$

BMI is classified as underweight (BMI < 18.5), normal weight (BMI 18.5–24.9), overweight (BMI 25-29.9) and obese (BMI ≥ 30) [13].

#### Postoperative nausea and vomiting (PONV):

A patient was considered to have had PONV if any nausea and or vomiting episodes occurred and rescue antiemetics were administered within 24 h of the postoperative period [2].

#### Time to compliant of PONV:

The time taken by the patients to compliant with nausea and or vomiting during the postoperative period in the PACU and or in the postoperative Surgery Ward. It is counted from the extubation time to the development of PONV within 24 h of the postoperative period.

### Data management

Data collectors, the nurses of PACU and Surgery Ward were trained on the study instrument, consent form, data collection procedure, and confidentiality of the respondents. The collected data were checked for completeness daily by the investigators to monitor the overall quality of the data collection process.

### Data analysis

Data were double-entered and validated using EpiData (Version 3.1 for entry and version 2.2.2.183 for analysis, EpiData Association, Odense, Denmark). Data were exported and analyzed using SPSS (version 23). *The Kolmogorov-Smirnov test* was performed to test for the normality of the study data. Normally distributed continuous variables of demographic and clinical characteristics were compared using a t-test. Non-normally distributed two independent variables were compared using the Mann-Whitney test and expressed as medians (IQR). The Chi-square or Fisher’s exact test was used to analyze categorical variables and presented in frequencies and percentages. Binary logistic regression analysis was performed to determine the factors associated with postoperative nausea and vomiting and odds ratio (OR) with a 95% confidence interval was computed to identify the strength of association with respective p-value < 0.05 declaring the presence of a significant association. Pearson’s correlation test was done for two quantitative variables to study the correlations.

### Ethics considerations

Ethical approval was granted by the Research Ethics Board of Health, Ministry of Health, Bhutan (Reference number: REBH/Approval/2017/072 dated 4/12/2017). Written informed consent was obtained from all the study participants.

## Results

There were 202 patients with cholelithiasis admitted in the Surgery Ward during the study period among which, 12 were excluded (converted to open cholecystectomy in 5, pregnancy in 4, and ASA > II in 3). Among 190 patients included in the study, 59 patients developed PONV, and the rate of PONV was 31.1% (59/190).

More than half (53.7%, 102/190) of the study population were within the 21–40 years of age range, over 80% (157/190) were female, and 2/3rd were overweight and or obese.

The most frequently prescribed premedication was ranitidine (39%, 34/87) and metoclopramide (31%, 27/87). Morphine was the most frequently used opioid analgesic before induction (57.4%, 109/190), and paracetamol was the most used adjuvant analgesic (83.6%, 51/61). Sodium thiopentone was a commonly used induction agent (65.8%, 125/190). Among the muscle relaxant agents, succinylcholine (45.1%) and atracurium (42.8%) were mostly preferred by the anesthesia personnel. Isoflurane (48.4%) and air (34.2%) were used in most of the patients as inhalation anesthetic agents for the maintenance of anesthesia. The most preferred antiemetic during the intraoperative period was ondansetron (65.9%). Neostigmine and atropine were used as reversal agents in all the patients. In more than half of the patients (52,6%, 100/190), the laparoscopic cholecystectomy surgery took more than 60 min. The IV fluid used intraoperative period was significantly less in patients with PONV as compared to those without PONV (666.9 ml vs. 763.5 ml, *p* < 0.05). The demographic variables were comparable between the patients without PONV and those with PONV (Table 1).

**Table 1:** Demographic characteristics, perioperative medications, and anesthetic agents used for the patients who were evaluated for postoperative nausea and vomiting following laparoscopic cholecystectomy at the National Referral Hospital, Bhutan, 2018

Demographic characteristics		Total *n* (%)	Postoperative nausea and vomiting	
			No (*n* = 131)	Yes (*n* = 59)
**Age (years)**				
	< 21	3 (1.6)	2 (1.5)	1 (1.7)
	21–40	102 (53.7)	66 (50.4)	36 (61.0)
	> 40	85 (44.7)	63 (48.1)	22 (37.3)
Median age (IQR)		39 (30–50)	40 (34–51)	36 (28–49) **
**Sex**				
	Male	33 (17.4)	26 (19.8)	7 (11.9)
	Female	157 (82.6)	105 (80.2)	52 (88.1)
**Body mass index (BMI)**				
	Normal	50 (26.3)	33 (25.2)	17 (28.8)
	Underweight	2 (1.1)	1 (0.8)	1 (1.7)
	Overweight	126 (66.3)	87 (66.4)	39 (66.1)
	Obese	12 (6.3)	10 (7.6)	2 (3.4)
**ASA classification**				
	ASA I	142 (74.7)	94 (71.8)	48 (81.4)
	ASA II	48 (25.3)	37 (28.2)	11 (18.6)
**Previous history**				
	History of surgery	39 (24.2)	33 (32.0)	6 (10.3)
	History of PONV	4 (2.5)	3 (2.9)	1 (1.7)
	History of motion sickness	64 (39.8)	28 (27.2)	36 (62.1)
	History of gastritis and or PUD	54 (33.5)	39 (37.9)	15 (25.9)
**Premedication**				
	Midazolam	19 (23.7)	13 (25.0)	6 (21.4)
	Ranitidine	34 (42.5)	20 (38.4)	14 (50.0)
	Metoclopramide	27 (33.8)	19 (36.6)	8 (28.6)
**Opioid analgesics**				
	Fentanyl	81 (42.6)	57 (43.5)	24 (40.7)
	Morphine	109 (57.4)	74 (56.5)	35 (59.3)
**Adjuvant analgesics**				
	Paracetamol	51 (83.6)	42 (89.4)	9 (64.3) *
	Diclofenac sodium	10 (16.4)	5 (10.6)	5 (37.7)
	Tramadol	0 (0.0)	0 (0.0)	0 (0.0)
**Induction agents used**				
	Sodium Thiopentone	125 (65.8)	75 (57.3)	50 (84.7)
	Propofol	65 (34.2)	56 (42.7)	9 (15.3)
	Ketamine	0 (0.0)	0 (0.0)	0 (0.0)
**Muscle relaxants**				
	Succinylcholine	156 (45.1)	106 (44.7)	50 (45.9)
	Vecuronium	42 (12.1)	25 (10.6)	17 (15.6)
	Atracurium	148 (42.8)	106 (44.7)	42 (38.5)
**Maintenance anesthetic agent**				
	Nitrous oxide	60 (15.8)	45 (17.2)	15 (12.7)
	Air	130 (34.2)	86 (32.8)	44 (37.3)
	Isoflurane	184 (48.4)	126 (48.1)	58 (49.2)
	Sevoflurane	3 (0.8)	2 (0.8)	1 (0.8)
	Halothane	3 (0.8)	3 (1.1)	0 (0.0)
**Intraoperative antiemetics used**				
	Dexamethasone	80 (32.5)	58 (31.7)	22 (34.9)
	Ondansetron	162 (65.9)	121 (66.1)	41 (65.1)
	Metoclopramide	4 (1.6)	4 (2.2)	0 (0.0)
**Duration of surgery (minutes)**				
	≤ 60	90 (47.4)	61 (46.6)	29 (49.2)
	> 60	100 (52.6)	70 (53.4)	30 (50.8)
**Intraoperative IV fluids used (ml)**				
	Mean IV fluid (± SD)	733.5 (± 300.4)	763.5 (± 286.1)	666.9 (± 322.5) *
**Reversal agents**				
	Neostigmine and atropine	190 (100.0)	131 (100.0)	59 (100.0)

*p-value < 0.05; ** p-value > 0.05; IQR: interquartile range; IV: intravenous fluid; PONV: postoperative nausea vomiting; PUD: peptic ulcer disease; SD: standard deviation

### Factors associated with PONV

Previous history of motion sickness was significantly associated with PONV (OR 5.8, 95%CI 2.9–11.2, *p* < 0.001). Female gender and underweight patients were likely to develop PONV, however, the association was not significant (*p* > 0.05). IV fluids used during the intra-operative period were found to significantly prevent the development of PONV (OR 0.9, 95%CI 0.9-1.0, *p* = 0.042) (Table 2).

**Table 2:** Factors associated with postoperative nausea and vomiting among patients who underwent laparoscopic cholecystectomy at the National Referral Hospital, Bhutan, 2018

Characteristics		B	S.E	Wald	OR (95% CI)		*p*-value		
**Age (years)**									
	< 21				Reference				
	21–40	0.1	1.2	1.9	1.1 (0.1–12.4)		0.944		
	> 40	-0.4	1.2	0.1	0.7 (0.6–8.1)		0.774		
**Gender**									
	Male				Reference				
	Female	0.6	0.5	1.8	1.8 (0.7–4.5)		0.184		
**Body mass index**									
	Normal BMI				Reference				
	Underweight	0.8	1.4	0.2	1.9 (0.1–32.9)		0.646		
	Overweight	-0.1	0.4	0.2	0.9 (0.4–1.7)		0.696		
	Obese	-0.9	0.8	1.3	0.4 (0.1–1.9)		0.254		
**ASA classification**									
	ASA I				Reference				
	ASA II	-0.5	0.4	1.9	0.6 (0.3–1.2)		0.162		
**Previous history of**									
	Surgery	-1.1	0.5	5.0	**0.3** (0.1–0.9)		**0.022**		
	PONV	-0.3	1.2	0.1	0.7 (0.1–7.2)		0.792		
	Motion sickness	1.8	0.3	26.2	**5.8** (2.9–11.2)		**< 0.001**		
	Gastritis and or PUD	-0.2	0.4	0.4	0.8 (0.4–1.6)		0.539		
**Intraoperative IV fluids used (ml)**									
	IV fluids	-0.0	0.0	4.1	**0.9** (0.9-1.0)		**0.042**		
**Duration of surgery (minutes)**									
	≤ 60				Reference				
	> 60	-0.1	0.3	0.1	0.9 (0.5–1.6)		0.741		

OR: odds ratio; PUD: peptic ulcer disease; IV: intravenous

### Association of antiemetics and anesthetic agents with PONV

Among the antiemetics used during the operation, ondansetron (OR 0.2, 95%CI 0.1–0.4, *p* < 0.001) was found to prevent the development of PONV by 80% as compared to dexamethasone and metoclopramide. Using a combination of more than two antiemetics from different classes significantly prevents PONV by 90% (OR 0.1, 95%CI 0.0-0.4, *p* = 0.002). Sodium thiopental used for the induction of anesthesia was 4 times more likely to develop PONV (OR 4.1, 95%CI 1.9–9.1, *p* < 0.001) as opposed to propofol which was found to prevent PONV. Muscle relaxants, opioid morphine, adjuvant analgesic diclofenac sodium, and sevoflurane and isoflurane used for maintenance of anesthesia were likely to develop PONV, however, the association was not statistically significant (*p* > 0.05). The details of the association of antiemetics and anesthetic agents are shown in Table 3.

**Table 3:** Association of antiemetics and anesthetic agents with postoperative nausea and vomiting among patients who underwent laparoscopic cholecystectomy at the National Referral Hospital, Bhutan, 2018

Characteristics		B	S.E.	Wald	OR (95% CI)	*p*-value
**Premedication**						
	Ranitidine	0.5	0.4	1.9	1.7 (0.8–3.7)	0.162
	Midazolam	0.7	0.5	2.4	2.0 (0.8–4.9)	0.123
**Induction of anesthesia**						
	Sodium thiopental	1.4	0.4	12.5	**4.1** (1.9–9.1)	**< 0.001**
	Propofol	-1.4	0.4	12.5	**0.2** (0.1–0.5)	**< 0.001**
**Antiemetics**						
	Metoclopramide	0.1	0.4	0.0	1.1 (0.5–2.4)	0.857
	Ondansetron	-1.7	0.4	14.8	**0.2** (0.1–0.4)	**< 0.001**
	Dexamethasone	-0.3	0.3	0.8	0.7 (0.4–1.4)	0.367
**Combination of antiemetics used**						
	One antiemetics used	-2.4	0.5	19.8	**0.1** (0.0-0.2)	**< 0.001**
	Two antiemetics used	-1.7	0.5	11.4	**0.2** (0.1–0.5)	**0.001**
	More than two antiemetics used	-2.4	0.8	9.6	**0.1** (0.0-0.4)	**0.002**
**Muscle relaxants**						
	Succinylcholine	0.4	0.4	0.6	1.4 (0.6–3.4)	0.433
	Vecuronium	0.5	0.4	2.2	1.7 (0.8–3.5)	0.137
	Atracurium	-0.5	0.3	2.2	0.6 (0.3–1.2)	0.137
**Opioids**						
	Morphine	0.1	0.3	0.1	1.1 (0.6–2.1)	0.715
	Fentanyl	-0.1	0.3	0.1	0.9 (0.4–1.6)	0.714
**Adjuvant analgesics**						
	Paracetamol	-0.9	0.4	4.9	**0.4** (0.2–0.8)	**0.026**
	Diclofenac sodium	0.6	0.7	0.9	1.8 (0.5–6.8)	0.343
**Maintenance of anesthesia**						
	Nitrous oxide	-0.4	0.4	1.5	0.7 (0.3–1.3)	0.222
	Sevoflurane	0.1	1.2	0.0	1.1 (0.1–12.5)	0.931
	Isoflurane	0.8	1.1	0.6	2.3 (0.3–20.1)	0.451
	Air	0.4	0.3	1.4	1.5 (0.7-3.0)	0.222
**Constant**		-0.4	0.2	4.9	0.7	0.026

OR: odds ratio

**Fig. 1:**
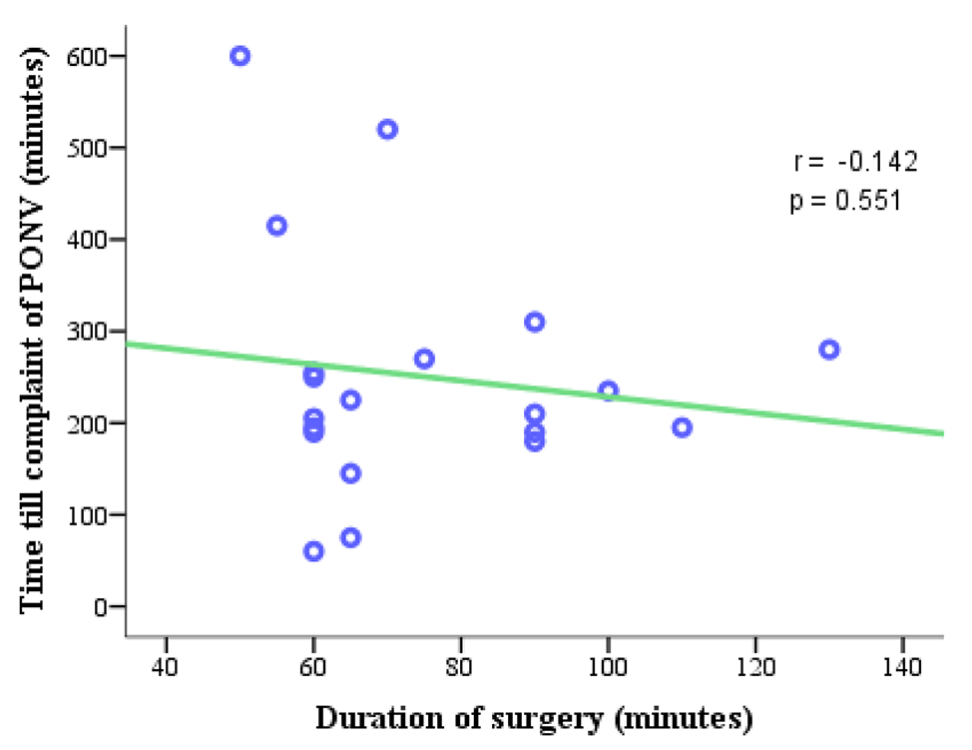
Scatter plot showing the correlation of duration of surgery (minutes) with the time taken for compliant of PONV

With the longer duration of surgery, it took less time to develop postoperative nausea and vomiting. There is a negative correlation between the duration of surgery and the time taken for compliant of PONV with *r* = − 0.14 and *p* = 0.551 (Fig. 1).

## Discussion

The rate of PONV after laparoscopic cholecystectomy under general anesthesia in this study was 31.1%. A slightly lesser prevalence (27.7%) of PONV was reported in a systemic review and meta-analysis performed on a total of 23 studies with 22,683 people who underwent laparoscopic cholecystectomy under general anesthesia from 11 countries [2]. The slightly higher rate of PONV reported in the current study is partly due to inadequate and or improper use of prophylactic antiemetics and anesthetic agents by the anesthesia personnel attending the surgery. The prescribing patterns of antiemetics differ from one anesthesia personnel to another due to the lack of a standard national guideline on the prevention and management of PONV in Bhutan [14].

Among many risk factors for PONV, laparoscopic surgery itself independently increases the risk of PONV. However, adequate and timely use of prophylactic antiemetics can prevent it [9]. As per the fourth consensus guidelines for the management of PONV, the use of multimodal antiemetic therapy which includes combining antiemetics from different drug classes is recommended to increase the effectiveness and prevent PONV [4]. Among the antiemetic agents available, the most frequently used antiemetics in the current study were ondansetron and dexamethasone. These two antiemetics were used in combination in some and used singly in a few patients.

Ondansetron is a selective 5-HT3 serotonin receptor antagonist that acts both centrally and peripherally to prevent and treat nausea and vomiting. Ondansetron acts centrally at area postrema (chemoreceptor trigger zone) which is located at the medulla oblongata in the brainstem and antagonizes the 5HT-3 receptor and prevents nausea and vomiting. Ondansetron also acts peripherally at the vagus nerve terminals and blocks 5-HT3 receptors available on the vagus nerves and prevents nausea and vomiting [10].

Dexamethasone is a glucocorticosteroid, when used in combination with other antiemetics, it has been reported to increase anti-emetic effects. The exact mechanism for the anti-emetic effect of dexamethasone is not completely understood, but it is thought to be caused by inhibiting the synthesis of prostaglandin and by causing a decrease in the release of endogenous opiates. Despite the obscured mechanism of action, dexamethasone is recommended to be used along with other drugs as prophylactic antiemetics for the prevention of PONV [4].

Another commonly used anti-emetics for the prevention of PONV is metoclopramide. It is a dopamine (D2) receptor antagonist. Metoclopramide inhibits D2 and 5-HT3 receptors at the chemoreceptor trigger zone in the central nervous system and at other organ systems and prevents nausea and vomiting.

In the current study, the use of ondansetron alone is found to prevent PONV by 80%, however, when the combination of more than two anti-emetics was used, it was found to prevent PONV by 90%. This finding is in line with the finding from other studies that a combination of anti-emetics of two or more is robust and superior over a single agent in preventing PONV [4].

Previous history of motion sickness and the use of sodium thiopental were found significantly associated with PONV in the present study. Other studies have reported females, with a history of PONV, surgery, and motion sickness as the independent risk factors for PONV [2, 15].

The use of propofol as an induction agent and paracetamol as an adjuvant analgesic is found to prevent PONV. A randomized controlled trial showed similar findings to the present study, with 37% and 72% reduction in the incidence of PONV in the groups who were administered dexamethasone and propofol respectively [16].

The duration of surgery and anesthesia is also a risk factor for the development of PONV. The risk of PONV increases with the increase in duration of anesthesia and surgery [17]. In the current study, those patients who underwent longer surgical procedures took less time to complain of nausea and vomiting during the postoperative period (Fig. 1). However, the logistic regression analysis showed duration of the surgery (> 60 min) is not associated with the development of PONV.

The use of adequate IV fluids intraoperative period is found to have preventive effects on PONV [18]. In the present study, the amount of IV fluids used is significantly less in the patients who developed PONV as compared to those who did not develop. During the laparoscopic cholecystectomy procedures, adequate hydration with IV fluids is recommended to prevent PONV [4].

### Limitations

This was a single-centre, hospital-based study assessing the rates and factors associated with postoperative nausea and vomiting. The intraoperative surgical complications might have occurred, which was not covered in this study, and this might have influenced the rates of postoperative nausea and vomiting.

## Conclusion

One-third of patients undergoing laparoscopic cholecystectomy under general anesthesia reported postoperative nausea and vomiting. Previous history of motion sickness and the use of sodium thiopental for the induction of anesthesia were significantly associated with PONV. This study re-emphasizes the importance of identifying high-risk patients and administration of appropriate antiemetic prophylaxis before induction of anesthesia to prevent postoperative nausea and vomiting. It is also imperative to have a standard national guideline on the prevention and management of postoperative nausea and vomiting, so the anesthesia personnel can maintain uniformity while prescribing antiemetics and reduce the untoward postoperative complications.

### Electronic supplementary material

Below is the link to the electronic supplementary material.

Supplementary Material 1

## Data Availability

The datasets used and/or analyzed during the current study are available from the corresponding author upon reasonable request.

## Abbreviations

ASA: American Society of Anaesthesiologists
BMI: Body mass index
CI: Confidence interval
IQR: Interquartile range
IV: Intravenous fluid
JDW: Jigme Dorji Wangchuk
OR: Odds ratio
PACU: Post Anesthesia Care Unit
PONV: Postoperative nausea and vomiting
PUD: Peptic ulcer disease
SD: Standard deviation
SPSS: Statistical package for the social sciences
