# Supplementary material for: Factors associated with postoperative nausea and vomiting after laparoscopic cholecystectomy at the National Referral Hospital, Bhutan: a cross-sectional study
Source: BMC Anesthesiol. 2024 Jul 22;24:248. doi: 10.1186/s12871-024-02602-w (PMC11265021; doi:10.1186/s12871-024-02602-w)
Supplement: Supplementary file 1 — Supplementary Material 1 [file 12871_2024_2602_MOESM1_ESM.docx]

| **Title: Factors associated with postoperative nausea and vomiting after laparoscopic cholecystectomy at the National Referral Hospital, Bhutan** | | | |
| --- | --- | --- | --- |
| **Data collection pro forma** | | |  |
| **Age** (years): ............................................................................................................................. | | | |
| **Gender:** | | |  |
|  |  | Male |  |
|  |  | Female |  |
| **BMI** | |  |  |
|  |  | Underweight |  |
|  |  | Normal |  |
|  |  | Overweight |  |
|  |  | Obese |  |
| **ASA** | |  |  |
|  |  | I |  |
|  |  | II |  |
| **Previous history of:** | | |  |
|  |  | Surgery |  |
|  |  | PONV |  |
|  |  | Motion sickness |  |
|  |  | Gastritis or PUD |  |
| **Premedication used:** | | |  |
|  |  | Midazolam |  |
|  |  | Ranitidine |  |
|  |  | Metoclopramide |  |
| **Opioids analgesics used:** | | |  |
|  |  | Fentanyl |  |
|  |  | Morphine |  |
| **Adjuvant analgesics used:** | | |  |
|  |  | Paracetamol |  |
|  |  | Diclofenac sodium |  |
| **Induction agents used:** | | |  |
|  |  | Sodium Thiopentone |  |
|  |  | Propofol |  |
|  |  | Ketamine |  |
| **Muscle relaxants used:** | | |  |
|  |  | Succinylcholine |  |
|  |  | Vecuronium |  |
|  |  | Atracurium |  |
| **Maintenance anesthetic agents used:** | | |  |
|  |  | Nitrous oxide |  |
|  |  | Air |  |
|  |  | Isoflurane |  |
|  |  | Sevoflurane |  |
|  |  | Halothane |  |
| **Intraoperative antiemetics used:** | | |  |
|  |  | Dexamethasone |  |
|  |  | Ondansetron |  |
|  |  | Metoclopramide |  |
| **Duration of surgery (minutes):…...........................................................................................** | | | |
| **Intraoperative IV fluids (ml):……...........................................................................................** | | | |
| **Reversal agents used:** | | |  |
|  |  | Neostigmine |  |
|  |  | Atropine |  |
| **Duration of surgery (minutes): ......................................................................................** | | |  |
| **Time to compliant of PONV during postoperative period (minutes):………..…………..........** | | | |
| **Postoperative events:** | | |  |
|  |  | Nausea |  |
|  |  | Vomiting |  |
|  |  | Others |  |
|  |  | None |  |
|  |  |  |  |
